# A retrospective study assessing the acceleration effect of type I *Helicobacter pylori* infection on the progress of atrophic gastritis

**DOI:** 10.1038/s41598-021-83647-6

**Published:** 2021-02-18

**Authors:** Weidong Liu, Junjie Tian, Wenjia Hui, Wenjie Kong, Yan Feng, Junqiang Si, Feng Gao

**Affiliations:** 1 Department of Gastroenterology, People’s Hospital of Xinjiang Uygur Autonomous Region, No. 91 Tianchi Road, Tianshan District, Urumqi, 83000 China; 2 Department of Physiology, Shihezi University of Medicine, Shihezi, China

**Keywords:** Gastritis, Gastritis, Bacterial host response

## Abstract

Based on the antibody typing classification, *Helicobacter pylori* infection can be divided into type I *H. pylori* infection and type II *H. pylori* infection. To observe the effects of different *H. pylori* infection types on the distribution of histopathological characteristics and the levels of three items of serum gastric function (PG I, PG II, G-17). 1175 cases from October 2018 to February 2020 were collected with ratio 1:2. All patients were performed with ^14^C-Urea breath test (^14^C-UBT), *H. pylori* antibody typing classification, three items of serum gastric function detection, painless gastroscopy, pathological examination, etc. According to *H. pylori* antibody typing classification, patients were divided into three groups: type I *H. pylori* infection group, type II *H. pylori* infection group and control group. Significant difference existed among type I *H. pylori* infection group, type II *H. pylori* infection group and control group in inflammation and activity (*χ*^2^ = 165.43, 354.88, *P* all < 0.01). The proportion of three groups in OLGA staging had statistic difference (*χ*^2^ = 67.99, *P* all < 0.01); Compared with type II *H. pylori* infection group and control group, the level of pepsinogen I, pepsinogen II, gastrin17 in type I *H. pylori* infection group increased, and PG I/PG II ratio (PG I/PG II ratio, PGR) decreased, which was statistically significant (*χ*^2^ = 35.08, 166.24, 134.21, 141.19; *P* all < 0.01). Type I *H. pylori* infection worsened the severity of gastric mucosal inflammation and activity. *H. pylori* infection was prone to induce atrophy of gastric mucosa, while type I *H. pylori* infection played a key role in promoting the progress of atrophic gastritis and affected the level of serum gastric function. The study indicated that the eradication of *H. pylori* should be treated individually.

## Introduction

*Helicobacter pylori* (*H. pylori*), a single polar, multiple flagellum and spirally curved gram-negative bacilli belongs to the micro aerobic bacteria, whose main infection site is the stomach and duodenal sphere. *H. pylori* has a significant correlation with chronic gastritis with gastric mucosal atrophy and erosion, peptic ulcer, MALT lymphoma and gastric cancer (GC)^1,2^. About 20% of *H. pylori* infected patients develop precancerous lesions but only 2% develop gastric cancer, which is closely related to the host genetic, environment and virulent strains of *H. pylori*^3^. *H. pylori* has different characteristics of virulence of the infecting strain. The pathogenesis of *H. pylori* may be associated with a variety of pathogenic factors, including cytotoxin associated protein A (cytotoxin associated protein A, CagA), vacuolating cytotoxin A (vacuolating cytotoxin A, VacA), duodenal ulcer promoting gene A and Adhesin, etc^4,5^. CagA and VacA are the two important determinants of *H. pylori* toxicity, inducing the damage of epithelial cells and chronic inflammation, which may ultimately lead to GC^6^. Based on the expression of serum virulence protein, the type of *H. pylori* infection is determined by antibody typing classification. If express CagA or VacA, it can be diagnosed as type I *H. pylori* infection, if not express CagA or VacA but Urease, it can be diagnosed as type II *H. pylori* infection.

Atrophic gastritis (AG) mainly caused by bad eating habits, smoking, drinking and *H. pylori* infection^7^. which is characterized by the decrease or disappearance of gastric acid glands, or accompanied by pylorus gland metaplasia and intestinal metaplasia (IM). A large sample cohort study has shown that *H. pylori* positive patients experienced chronic gastritis to AG and eventually to intestinal metaplasia, and the risk of gastric cancer is also at a staircase increase^8^. Serological tests, including gastrin 17 (G-17) and pepsinogen (PG), could help to identify individuals at risk for AG. PG is an inactive precursor of pepsin in gastric juice and a special biological marker of gastric secretion. According to immunological activity and biochemical characteristics, PG can be divided into two categories: PGI and PGII, the former is mainly secreted by the chief cell and mucous neck cells, while the latter is secreted by fundus gland, cardiac glands, pyloric glands and Brunner's glands. Most of the synthetic PG is released into the gastric cavity, and only about 1% enter the blood circulation^9^. In ideal state, serum PG can accurately reflect the state of gastric mucosal lesion^10–12^. G-17 is an important gastrointestinal peptide hormone and produced in the antrum by G cells, which is correlated with the position and degree of gastric mucosal atrophy. When the synthesis of G-17 decreases, it could indicate the presence of AG in the antrum, while the presence of AG in the corpus can led to the increase of G-17 by the negative feedback of gastrin gastric acid axis^13^. This study aims to analyze the relationship between different types of *H. pylori* infection on the distribution of histopathological characteristics and the levels of three items of serum gastric function. As well as to provide a theoretical basis for the clinical individual eradication of *H. pylori* and a possible mechanism of type I *H. pylori* infection in the development of AG.

## Methods

### Subject investigated

In this retrospective analysis, 1175 inpatients were recruited in department of gastroenterology of people’s hospital of Xinjiang Uygur Autonomous Region from October 2018 to February 2020. All inpatients were performed with ^14^C-Urea breath test (^14^C-Urea breath test, ^14^C-UBT), *H. pylori* antibody typing classification, three items of serum gastric function detection (PGI, PGII, G-17), painless gastroscopy, pathological examination, etc. Exclusion criteria: ethnic minorities; History of previous gastric cancer surgery; Result of ^14^C-UBT and *H. pylori* antibody typing classification was not conformed. Based on *H. pylori* antibody typing classification, all inpatients were divided into three groups: type I *H. pylori* infection group, type II *H. pylori* infection group and control group. 235 inpatients were enrolled in type II *H. pylori* infection group and the other two groups were 470 inpatients respectively (1:2 paired). Written and informed consent was obtained from each participant older than 18 years old. The results of the test will be presented anonymously, which complied with the principles of the Declaration of Helsinki. This study also approved by the ethics committee of people’s hospital of Xinjiang Uygur Autonomous Region (KY2019051528).

### ^*14*^*C-UBT*

Inpatient took a ^14^C Urea capsule (0.75 μci) on an empty stomach and sat quietly for 25 min, then blew bubbles to the gas collecting bottle until the color of the liquid indicator changed from pink to colorless. Added 4.5 ml scintillation solution to the gas collecting bottle, reversed three times and put into the *H. pylori* detector (HUBT-01A, headway) then the detection time set at 1 min. The detection value ≥ 100 dpm was identified to be *H. pylori* positive and the value < 100 dpm was identified to be negative.

### *H. pylori* antibody typing classification

Fasting venous blood 2–3 ml with a procoagulant tube was collected and separated. *H. pylori* antibody typing classification assay kit (western blotting) was provided by Shenzhen bolt biotech Co., Ltd. Experimental process through serum antibody combined with dot blot membrane, enzyme-linked reaction, chromogenic reaction and terminal reaction, and the result determined by the standard band. According to *H. pylori* antibody typing classification, *H. pylori* infection was divided into type I *H. pylori* infection group, type II *H. pylori* infection group and control group. If expressed CagA or VacA, it could be diagnosed as type I *H. pylori* infection. If not expressed CagA and VacA but Urease A or Urease B, it could be diagnosed as type II *H. pylori* infection. If only expressed the quality control, which could be diagnosed as *H. pylori* negative. The appearance of band of quality control did not indicate that the test was invalid.

### Three items of serum gastric function detection

Fasting venous blood 2–3 ml was collected by vacuum tube, then the serum was separated. PGI, PGII, G-17 test kit (Fluorescence immunochromatographic assay) was provided by BIOHIT Healthcare (Hefei) Co., Ltd. Adding 80 μl serum to the test kit and placing in the Fluorescence immunoquantitative analyzer (HIT-91A) 15 min later. The results could be read by fast detection. The normal reference range of PGI, PGII, PGR and G-17 was 70–165 μg/l, 3–11 μg/l, 7–20 and 1–7 pmol/l, respectively.

### Gastroscopy and pathological diagnosis

The diagnostic criteria for gastroscopy and pathology were based on Chinese consensus on chronic gastritis (2017, Shanghai)^14^. Five gastric mucosa biopsies were taken according to the standard, including two gastric antrum biopsies, two gastric body biopsies and one gastric corner biopsy. Histopathological evaluation was performed according to the Sydney system. Chronic inflammation, activity, atrophy and intestinal metaplasia were graded as absent (0); mild (1); moderate (2) and severe (3).

### Statistical analysis

SPSS17.0 software was used to analyze the data. Continuous variables were described as mean (± standard deviationand), while categorical variables were described as percentages or frequencies, while categorical variables were described as percentages or frequencies. All data were tested for normal distribution by Shapiro–Wilk test. Kruskal–Wallis test was used to compare multiple quantitative variables, while chi-square test or Mann–Whitney U were used for pairwise comparison between groups. *P* value less than 0.05 was considered statistically significant.

## Results

### General information

According to the 1:2 paired conditions with age and sex, 235 inpatients were enrolled in type II *H. pylori* infection group and the other two groups were 470 inpatients respectively. Among the 1175 inpatients, there were 630 males and 545 females, aging from 30 to 82 years old, with an average age of 55.9 ± 9.4 years. There were 470 cases in type I *H. pylori* infection group, 252 males and 218 females, with an average age of 56.1 ± 9.1 years; 235 cases in type II *H. pylori* infection group, 126 males and 109 females, with an average age of 56 ± 9.5 years; and 470 cases in control group, 215 males and 212 females, with an average age of 55.7 ± 9.6 years. No significant differences in sex and age among the three groups (*χ*^2^ = 0, *P* = 1; *χ*^2^ = 1.69, *P* = 0.43, *P* all > 0.05).

### The relationship between different types of *H. pylori* infection with inflammation and activity

The proportion of different types of *H. pylori* infection in inflammation and activity was shown in Table 1. In the distribution of inflammation degree, the proportion of moderate and severe inflammation in type I *H. pylori* infection group was higher than that in type II *H. pylori* infection group and control group (*χ*^2^ = 165.43, *P* all < 0.01). In the distribution of activity degree, the proportion of mild and moderate activity in type I *H. pylori* infection group was higher than that in type II *H. pylori* infection group and control group (*χ*^2^ = 176.22, *P* all < 0.01).

**Table 1:** The proportion of different types of *H. pylori* infection in inflammation and activity.

Group	Type I *H. pylori* infection group	Type II *H. pylori* infection group	Control group	*χ*^2^	*P* value
*n*	470	235	470		
**Inflammation**					
0	9 (1.9%)	5 (2.2%)	25 (5.3%)	350.67	< 0.01
1	139 (29.6%	162 (68.9%)	394 (83.8%)		< 0.01
2	306 (65.1%)	68 (28.9%)	51 (10.9%)		< 0.01
3	16 (3.4%)	0 (0%)	0 (0%)		< 0.01
**Activity**					
0	153 (32.5%)	178 (75.7%)	421 (89.6%)	354.88	< 0.01
1	217 (46.2%)	45 (19.2%)	41 (8.7%)		< 0.01
2	95 (20.2%)	12 (5.1%)	8 (1.7%)		< 0.01
3	5 (1.1%)	0 (0%)	0 (0%)		< 0.01

### Relationship between different types of *H. pylori* infection in OLGA staging

The histopathological evaluation of gastric mucosa biopsies was performed by updated Sydney system and the staging was expressed according to OLGA classification systems. In type I *H. pylori* infection group, only two inpatients with stage-3 OLGA staging, which were included in stage-2 OLGA staging for statistics facilitate. As it depicted in Table 2, the proportion of stage-2 in type I *H. pylori* infection group was higher than that in type II *H. pylori* infection group and control group, the difference had statistically significant (*χ*^2^ = 44.34, *P* all < 0.01). Stage-0 in control group, type II *H. pylori* infection group and type I *H. pylori* infection group increased in turn, the difference was statistically significant (*χ*^2^ = 67.99, *P* all < 0.01).

**Table 2:** The proportion of different types of *H. pylori* infection in OLGA staging.

Group	Type I *H. pylori* infection group	Type II *H. pylori* infection group	Control group	*χ*^2^	*P* value
*n*	470	235	470		
Stage-0	224 (47.7%)	127 (54%)	339 (72.1%)	67.99	< 0.01
Stage-1	197 (41.9%)	97 (41.3%)	114 (24.3%)		
Stage-2	49 (10.4%)	11 (4.7%)	17 (3.6%)		

### The effect of different types of *H. pylori* infection on three items of serum gastric function

Table 3 had depicted the effect of different types of *H. pylori* infection on serum three items of serum gastric function. Compared with type II *H. pylori* infection group, in type I *H. pylori* infection group, the level of PGI, PGII and G-17 increased and the PGR decreased, the difference was statistically significant (*Z* = − 5.52, − 11.97, − 11.52, − 10.69, respectively; *P* all < 0.01). Compared with the control group, there was no significant difference in the level of PGI, PGII, PGR (*Z* = − 0.54, − 0.56, − 0.35; *P* all > 0.05), only the level of G-17 increased (*Z* = − 2.31, *P* < 0.05).

**Table 3:** The effect of different types of *H. pylori* infection on three items of serum gastric function.

Group	Type I *H. pylori* infection group	Type II *H. pylori* infection group	Control group	*χ*^2^	*P* value
*n*	470	235	470		
PGI	186.4 ± 87.8	159.8 ± 77.3	159.6 ± 80.6	35.08	< 0.01
PGII	11.4 ± 69.2	6.36 ± 5.1	6.6 ± 6.7	166.24	< 0.01
PGR	22.8 ± 16.9	32.9 ± 23.3	32 ± 17.5	134.21	< 0.01
G17	9.9 ± 12.7	6.2 ± 10.9	5.6 ± 11.4	141.19	< 0.01

## Discussion

It is a consensus that *H. pylori* infection is the cause of chronic gastritis, and *H. pylori* can cause sustained damage to gastric mucosa. *H. pylori* is viewed as a key cause in many diseases, such as chronic gastritis with atrophy and erosion, peptic ulcer, MALT lymphoma, gastric cancer and so on. AG is considered to be the outcome of *H. pylori* infection and is associated with a high risk of gastric cancer^15–18^. Although eradication of *H. pylori* can reduce the risk of gastric cancer in the general population, the risk of gastric cancer do exist, which is associated with the degree of AG and IM. Histological studies showed that there is no significant change in AG and IM during the 2 years after eradication of *H. pylori*, and AG and IM are significantly improved 10 years later^19^.

Eradicating of *H. pylori* is not always beneficial to some diseases, because it can lead to an increase in the level of gastric acid secretion, which may increase gastroesophageal reflux and ulceration of low dose aspirin. Therefore, individualized eradication of *H. pylori* is the best way^20^. OLGA classification and serum gastric function detection are optimal for early gastric cancer screening. A surveillance program including OLGA stage and serum gastric function status may facilitate early detection of gastric cancer^21^. ACG Clinical Guideline have demonstrated that patients over 60 years old and unexplained dyspepsia with no early warning symptoms are recommended for non endoscopic *H. pylori* tests, and those with positive results should be treated with eradication^22^. *H. pylori* infection is one of the most important factors causing atrophy and precancerous lesions. Recently, countries around the world are actively eradicating *H. pylori* infection to reduce the incidence rate of gastric cancer. However, only few countries can eradicate *H. pylori* infection for all, indicating that individualized treatment is the feasible strategy.

*H. pylori* has a variety of diagnostic methods^23,24^. The most non-invasive detection methods are ^14^C-UBT and serum *H. pylori* antibody typing classification. There may be false positive and false negative results in ^14^C-UBT. The combination of the two test results can accurately estimate *H. pylori* infection^25^. *H. pylori* antibody typing classification is mainly based on the expression of virulence protein CagA, VacA, Urease A and Urease B. CagA of *H. pylori* can accelerate the progress of gastric cancer and AG, in which tyrosine phosphorylated EPIYA modules play an important role in active gastritis, associating with the progression of diffuse-type of gastric cancer, especially in women^26^. The meta-analysis has summarized 33 studies and points that *H. pylori* CagA genotypes are associated with increased risk of AG, IM, and gastric cancer, especially IM and gastric cancer^27^. CagA positive *H. pylori* can downregulation of autophagy in the gastric mucosa and induce the increase of inflammatory factors and increase the degree of gastritis^28^. Through different types of *H. pylori* infection and histopathological characteristics analysis, it found that the degree of inflammation and activity caused by type I *H. pylori* was significantly higher than that caused by type II *H. pylori.* According to OLGA classification systems, histopathological evaluation results showed that type I *H. pylori* tended to a higher staging levels. Thus, it is speculated that *H. pylori* infection and its pathogenicity are closely related to the specific genes and proteins. The results suggested that different types of *H. pylori* infection had different characteristics, as well as explained that most of the patients with *H. pylori* infection did not have any symptoms.

In the gastric epithelial cells, CagA is transmitted through a unique type IV secretory system to the initial immune response, followed by a series of responses to recruit immune cells to form chronic gastritis, and serum CagA and VacA virulence proteins increase the risk of non cardia cancer, while the risk of low virulence protein of *H. pylori* is lower^29,30^. Serological detection of pepsinogen PG I, PGII, PGR and G-17, the epidemiological markers for gastric cancer risk investigation, has provided valuable information for the status of gastric mucosa^31^. This study showed that type I *H. pylori* infection could increase the level of serum PGI, PGII and G-17, possibly because the inflammatory mechanism. The study of *H. pylori* virulence factors were focused on the gene level, depicting that *CagA* and *VacA* gene positive samples have strong correlation with AG and IM, and can better predict AG and IM^32,33^. Some studies have found that the invasion ability of *H. pylori* infection is associated with the middle region of the *vacA* gene^34,35^. This result suggested that different types of *H. pylori* infection might be related to the size of the invasiveness. According to the different secretory sites of PGI and PGII in the stomach, it is speculated that type I *H. pylori* infection could increase the secretion of serum PG in the whole stomach and duodenum region. The infection of type I *H. pylori* infection might accelerate the progress of atrophic gastritis due to the increase the invasion of gastric mucosa and the level of serum gastric function through unique inflammatory mechanisms.

In conclusion, different types of *H. pylori* had different characteristics. Through the analysis of histopathological characteristics and serum gastric function, it is confirmed that type I *H. pylori* infection played a key role in worsening the progress of atrophic gastritis, while the role of type II *H. pylori* infection is relatively weak. Thus, the clinical application of *H. pylori* eradication should be differentiated based on the *H. pylori* antibody typing classification and individualizing eradication of *H. pylori* can benefit more people.
